# Gender differences in health in Havana versus in Mexico City and in the US Hispanic population

**DOI:** 10.1007/s10433-020-00563-w

**Published:** 2020-03-16

**Authors:** Mine Kühn, Carlos Díaz-Venegas, Domantas Jasilionis, Anna Oksuzyan

**Affiliations:** 1 Max Planck Institute for Demographic Research, Konrad-Zuse Straße 1, 18057 Rostock, Germany; 2 Demographic Research Centre, Vytautas Magnus University, Jonavos Str. 66-212, 44191 Kaunas, Lithuania

**Keywords:** Self-reported health, Physical disability, Depression, Female disadvantage, Cuba

## Abstract

Health progress in the 1960s and 1970s placed Cuba at the vanguard of longevity in Latin America and the Caribbean. This success has often been attributed to equity of access to the health care system and its cost-effectiveness in the country. Cuba also has a small gender gap in life expectancy. In this study, we examined how this pattern is reflected in the gender differences in health among the population aged 60+ in Havana. We compared gender differences in health in samples drawn from Havana, Mexico City, and the US Hispanic population: three geographic settings with very different political, health care, and social systems. The data come from the Survey on Health, Well-Being, and Aging in Latin America and the Caribbean and the 2000 Health and Retirement Study. Age-adjusted prevalence and logistic regressions were estimated for poor self-rated health, limitations on activities of daily living, depression, and mobility limitations. While an absolute female disadvantage in health was apparent in all three populations, the relative gender differences were inconsistent across all four health domains. Gender differences were most pronounced in Havana, even after adjusting for age, socio-economic status, family characteristics, and smoking behaviour. Despite having higher overall life expectancy and more equitable and universal access to primary care and preventive medicine, women in Havana appear to have a larger burden of ill health than women in less equitable societies. The study provides indirect evidence that Cuba faces challenges in combating the health threats posed by chronic diseases and other diseases and conditions common among the population aged 60+.

**Electronic supplementary material:**

The online version of this article (10.1007/s10433-020-00563-w) contains supplementary material, which is available to authorized users.

## Introduction

Empirical analyses have consistently shown that women live longer than men in almost all nations, although the magnitude of the gender gap varies across countries (Clark and Peck 2012). There is also evidence that women tend to show worse health. Substantial female disadvantages have been found in physical capability test outcomes (Keevil et al. 2013), rates of depression (Salk et al. 2017), and comorbidities (Case and Paxson 2005; Crimmins et al. 2011). A variety of studies have reported generally higher prevalences of poor self-perceived health (Crimmins et al. 2011) and functional limitations among women than among their male counterparts from very young to old ages (Palacios-Ceña et al. 2012).

Cross-country comparison studies enable researchers to investigate whether gender differences are universal across various health measures, but only a few studies have focused on gender differences in health in low- and middle-income countries, partly due to the lack of availability of high-quality, comparable survey data. Literature on the potential determinants of gender differentials in health in developing countries, and particularly in Latin America, has been scarce (Zunzunegui et al. 2015). To help fill this research gap, in this study we compared the gender differentials in health for populations aged 60+ in Havana (Cuba) and Mexico City (Mexico) and the population of older foreign-born US Hispanics.

These populations encompass three geographic settings with very different political, health care, and social systems. Cuba seems to be a special case among the Latin American countries: despite economic hardships, health outcomes in Cuba are similar to those in high-income countries (Drain and Barry 2010). World Health Organization (WHO) estimations for 2016 for Cuba showed a total life expectancy (LE) at birth of 79.0 years, which was very close to the average LE at birth in the USA (78.5 years) and slightly below that of Canada (82.8 years) (WHO 2016). The Cuban health system has succeeded in reducing infant mortality, increasing vaccination rates (MacDonald et al. 2006a), and eradicating poliomyelitis (Lago 1999). In 2001–2005, the neonatal, infant, and under-five child mortality rates were two to three times lower in Cuba than in Mexico and similar to estimates for the USA (WHO 2016). Cuba has been also successful in promoting medical education and implementing highly proactive primary (family) health care (Cooper et al. 2006). Success in health promotion has often been attributed to equity of access to the health care system and its strong emphasis on prevention and cost-effectiveness (Drain and Barry 2010).

At the same time, the Cuban population is ageing rapidly (Destremau 2019), which is likely to result in a growing population of older people with disabilities—a development that will pose huge challenges for the country (Destremau 2019; Durán Gondar and Chávez Negrín 2000). Although health care reforms were implemented with the goal of improving access to health care for the old-age population (e.g. the establishment of day-care facilities), these changes appear to be insufficient to meet health care demands in the light of the increasing incidence of major chronic conditions, such as cancer, cardiovascular diseases, obesity, and diabetes (Destremau 2019).

Mexico is one of the leading economies in Latin America but has contrasting outcomes with respect to health and survival. On the one hand, the country’s life expectancy has increased from 33.9 years in 1930 to 75.2 years in 2016, while on the other hand, Mexico still deals with infectious and parasitic diseases that tend to be more prevalent in rural areas (INEGI 2016).

In the USA, Hispanics are the largest racial/ethnic group after non-Hispanic whites. Overall, the population aged 65+ is increasing rapidly in the USA and is projected to reach nearly 100 million by 2060 (Mather et al. 2015). The population aged 65+ also faces substantial structural changes, with the share of Hispanics in this population expected to grow from 7% in 2010 to over 18% in 2050 (Ortman et al. 2014). Mexicans make up the largest share of the US Hispanic population (58.5%), while Cubans account for a much smaller percentage (3.5%). However, Cubans are, on average, the oldest group among all US Hispanics (median age of 40.7 years vs. 24.2 for Mexicans) (Guzmán 2001).

Hispanics in the USA have been extensively studied for the past three decades, in part because despite their socio-economic disadvantages, they have enjoyed better health and survival than non-Hispanic whites, a concept known as the Hispanic paradox (Markides and Coreil 1986; Markides and Eschbach 2005; Mehta et al. 2016). Scholars have hypothesised that the health and survival advantages observed among Hispanic migrants are attributable to the selection of healthier individuals into migration and that these advantages are reinforced by healthier lifestyle behaviours among migrants, including lower levels of smoking (Lariscy et al. 2015) and alcohol use (Jayaweera and Quigley 2010), as well as healthier diets (Dixon et al. 2000).

Over this period, the profile of the migrants entering the USA changed. In the late 1980s and the 1990s, young men with little or no education dominated migration to the USA. However, in the 2000s, the typical migrant had become a slightly older individual with a few more years of educational attainment. Although the majority of these migrants were men, the share of women was increasing (Sáenz 2015). The changes in the socio-demographic profiles of Hispanics immigrating to the USA have led to their more diverse health profiles (Bostean 2013). There is evidence that Hispanic women are at a much higher risk of developing depressive symptoms than women of any other race/ethnicity in the USA (Gonzales et al. 2006) and that both Hispanic men and women are at a higher risk of having a disability than non-Hispanics (Hayward et al. 2014). Thus, studying also the US Hispanic population enabled us to observe gender differences in health between populations who live in their countries of origin and populations who have settled in the USA.

### Gender differences in life expectancy (LE)

**Fig. 1:**
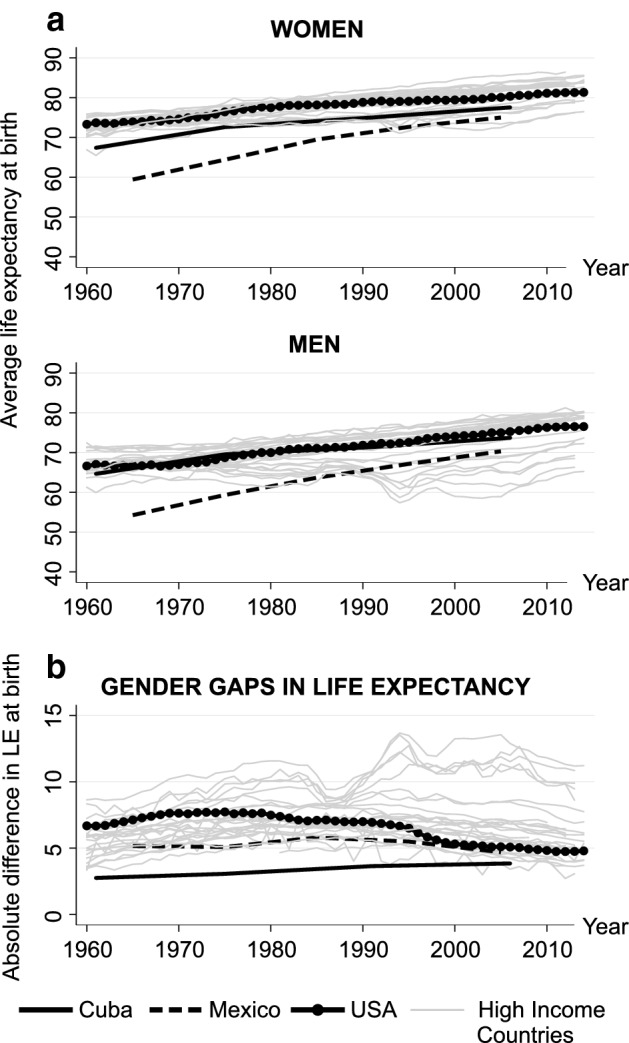
Female and male life expectancy and gender differences in life expectancy in Cuba versus in Mexico and in the USA and other high-income countries

Based on the Human Mortality Database (HMD) and the Latin American Mortality Database (LAMBdA) (Palloni et al. 2014), Fig. 1a shows that the LE at birth of Cuban men and women became comparable to that of their counterparts in high-income countries after 1970. In 2006, male LE in Cuba (73.7 years) was very close to that in the USA (75.3 years), while the difference in the LE was larger between women in Cuba (77.6 years) and women in the USA (80.4 years) and other developed countries. In the same year, the LE at birth was higher among US Hispanic women (82.9 years) and men (77.5 years) than in the US population as a whole.^1^ Thus, the absolute female–male differences in LE were smaller in Cuba (from 2.8 years in 1961 to 3.8 years in 2006) than in most developed countries (e.g. in the USA, from 6.7 years in 1960 to 5.1 years in 2006), among US Hispanics (5.4 years in 2006), and in Mexico (from 5.2 years in 1965 to 5.0 in 2005) over the whole period (Fig. 1b).

In the present study, we first asked whether the small gender gap in LE in Cuba is reflected in gender differences in four major health domains: self-rated health, activities of daily living, depression, and mobility limitations. Given the less pronounced LE improvements among Cuban women than among Cuban men, we expected to find a greater health advantage for men and, therefore, a larger gender gap in health in the Havana than in the Mexico City sample. Considering that the majority of the US Hispanics in the study population were Mexicans, and taking into account the healthy migrant effect and the predominantly male migration from Latin American countries to the USA, we hypothesised the gender differences in health in the US Hispanic population to be larger than in the Mexico City sample and comparable in magnitude to the Havana sample. Second, we identified to what extent the observed gender differentials in health in the three populations can be explained by socio-economic characteristics and smoking behaviours.

## Materials and methods

### Data sources

The data used come from the Survey on Health, Well-Being, and Aging in Latin America and the Caribbean (SABE) and from the Health and Retirement Study (HRS). The SABE was conducted during 1999 and 2000 to examine the health conditions and functional limitations of persons aged 60 or older. It is a cross-sectional, household-based study that covers seven cities in Latin America and the Caribbean: Buenos Aires, Argentina; Mexico City, Mexico; Santiago, Chile; Havana, Cuba; Montevideo, Uruguay; Bridgetown, Barbados; and São Paulo, Brazil (Pelaez et al. 2004). The HRS is a nationally representative panel of Americans aged 50 or older that was initiated in 1992. It includes responses from in-person and telephone interviews of more than 15,000 individuals. We used data from the year 2000 follow-up (Health and Retirement Study 2003) to match the year when the SABE was conducted.

### Study sample

The study sample consisted of 1905 SABE respondents from Havana (63% women) with a response rate of 95%, 1247 SABE respondents from Mexico City (59% women) with a response rate of 85%, and 561 HRS respondents who indicated that they were a foreign-born individual of Hispanic origin (57.2% women) with a response rate of 88%. The respondents in all three samples were aged 60 or older. (See Supplementary Table 1 for background characteristics.)

### Health and functional outcome variables

The global self-rated health (SRH) question asked interviewees to evaluate their health in general (“How do you consider your health in general?”) with five possible responses in all surveys: “excellent”, “very good”, “good”, “fair”, and “poor”. SRH was dichotomised with the response option “poor” as one and the other response options as zero. SRH is the most frequently used indicator of health in social, economic, and epidemiological research, as it is a strong predictor of mortality and disability (Idler and Benyamini 1997; Mossey and Shapiro 1982). Research has determined that SRH is a useful tool to measure the health of the Hispanic population, but the translation of some words may lead to worse ratings of SRH in Spanish than in English (Viruell-Fuentes et al. 2011).

Limitations on Activities of Daily Living were assessed using a modified version of the original ADL Index proposed by Katz et al. (1963), including the activities dressing, bathing, eating, getting in and out of bed, and toileting. The modified version excludes continence as it has been shown to be an imprecise instrument to quantify disability in older adults (Al Snih et al. 2009). While psychometric properties of the original scale were not formally tested, this index has been widely used in research (Shelkey and Wallace 1999). The response categories were “has difficulties”, “cannot do it”, “no difficulties”, and “does not do it”. Disability within each item was defined as the inability to perform the activity completely by oneself. A respondent who answered that he/she “cannot do” or “does not do” an activity was considered to have disability only if he/she needed help performing the activity. Respondents with one or more limitations in these activities were assumed to have an ADL disability.

For measuring the presence of depressive symptoms, the SABE and the HRS used two different sets of questions: the Geriatric Depression Scale (GDS) was applied in the SABE and the Centre for Epidemiologic Studies of Depression (CES-D) scale was applied in the HRS. The GDS consists of 15 items with dichotomous yes/no responses. The cut-off point used to identify symptoms of depression is six (Yesavage et al. 1982). The CES-D consists of an abbreviated version of the original scale with eight dichotomous yes/no responses to common symptoms. The cut-off point used to identify depression is three (Han 2002). Both the GDS and the CES-D have been deemed a valid and reliable measure of depressive symptoms among older adults (Martínez-De La Iglesia et al. 2002; Steffick 2000).

Mobility limitations were defined as having difficulties with or being unable to perform at least one of the following physical activities: walking several hundred yards, climbing several flights of stairs, and lifting weights over 5 kilograms (Rosow and Breslau 1966). While we are not aware of established validation of these items in English or Spanish, a Danish study demonstrated that the scale constructed using these three items and additionally the item dealing with the ability to run had high reliability in both the male and female samples for the in-person and proxy interviews (Christensen et al. 2002).

### Control variables

Age was measured as a categorical variable with four possible values (60–64, 65–69, 70–74, 75 and older). Educational attainment was measured by asking respondents the highest level of schooling they achieved (no education or incomplete primary, completed primary education, and some secondary or more) in the SABE and the total years they spent in education in the HRS. To make these categorisations comparable, we grouped the years spent in education from the HRS together with the equivalent levels of schooling achieved to match SABE. Income was measured by the respondents’ perceptions of their current income (sufficient, insufficient) in the SABE, and using four monthly income categories (no income or indebted as the reference category, earning less than $10,000 USD, earning between $10,000 and $19,999 USD, and earning $20,000 USD or more) in the HRS. Additional controls included current partnership (married/cohabitating, single/separated/divorced, and widowed), number of children (0, 1 or 2, 3 and more), and smoking status (never smoked, current smoker, and ex-smoker).

### Statistical analysis

First, we estimated age-standardised prevalences of poor SRH, ADL disability, depression, and mobility limitations for women and men using four age groups: 60–64, 65–69, 70–74, and 75 and older. We used the 2010 WHO World Population data as the reference population for our calculations (http://seer.cancer.gov/stdpopulations/world.who.html). Age standardisation was carried out as crude rates are not comparable across populations if the age composition of these populations is different (Ahmad et al. 2001). Second, to examine gender differences in SRH, ADL disability, depression, and mobility limitation we used logistic regression models including gender and controlling for age (Model I). Other controls were entered stepwise for each outcome. Additional to gender and age, we controlled for education and income (Model II), partnership status and the number of children (Model III), and smoking behaviour (Model IV). The estimates are presented as odds ratios (OR) with 95% confidence intervals (CI). We additionally considered p values and flagged coefficients according to their levels of significance (*p < 0.05, **p < 0.01, ***p < 0.001). All the analyses were conducted using Stata version 14.2 (StataCorp 2015).

## Results

**Fig. 2:**
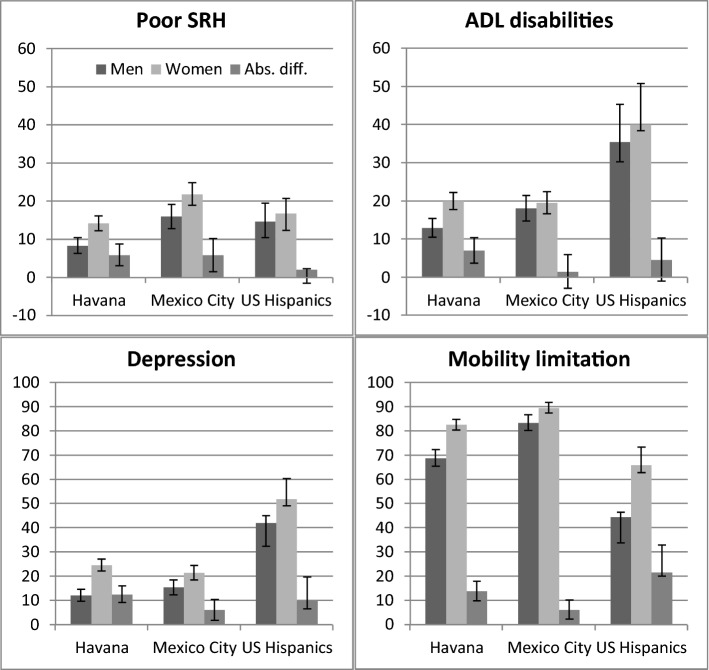
Age-standardised prevalences and absolute gender differentials in the prevalence of poor SRH, ADL disability, depression, and mobility limitations in Havana, Mexico City, and the US Hispanic population

Figure 2 shows the age-standardised prevalences of poor SRH, ADL disability, depression, and mobility limitations among men and women, and the absolute differences in these prevalences between men and women in Havana, Mexico City, and for the foreign-born Hispanics in the USA. There were notable variations in the magnitude and the significance of the absolute gender differentials. Women in all three samples had higher prevalences of poor SRH, ADL disability, depression, and mobility limitations than men. The absolute gender differences in poor SRH were similar in Havana (5.9% CI 3.03; 8.7) and Mexico City (5.9% CI 1.5; 10.23) and larger than among foreign-born US Hispanics (2.1%, CI 0.37; 3.81). The Havana sample showed the largest absolute gender differences in ADL disability (7.1% CI 3.71; 10.41) and depression (12.4% CI 9.03; 15.87) (versus 1.5% CI − 2.9; 5.89 and 6.1% CI 1.77; 10.39 in Mexico City; 4.6% CI 2.01; 7.11 and 9.9% CI 6.33; 13.39 for foreign-born US Hispanics), while the foreign-born US Hispanics had the largest absolute gender gap in mobility limitation (21.5% CI 16.62; 26.42 vs. 13.8% CI 9.74;17.81 in Havana; 6.1% CI 2.25; 10.09 in Mexico City) (Fig. 2).

Table 1 shows the odds of being in poor SRH, and having ADL disability, depression, and mobility limitations among women relative to men in each sample, while the relative differentials across categories of all control variables are presented in Supplementary Tables 2–5.

**Table 1:** Relative sex differentials (odds ratios) in poor SRH, ADL disability, depression, and mobility limitations in Havana, Mexico City, and the US Hispanic population

	Model I			Model II			Model III			Model IV		
	OR	CI 95%		OR	CI 95%		OR	CI 95%		OR	CI 95%	
*Poor self-rated health*												
Havana	1.84***	1.34	2.53	1.84***	1.33	2.55	1.85**	1.30	2.64	2.00***	1.38	2.92
Mexico City	1.47*	1.10	1.97	1.40*	1.03	1.89	1.44*	1.04	1.99	1.43	0.98	2.07
Foreign-born US Hispanics	1.21	0.77	1.92	0.99	0.62	1.60	1.03	0.64	1.68	1.14	0.66	1.83
*ADL disability*												
Havana	1.75***	1.35	2.27	1.66***	1.27	2.17	1.86***	1.39	2.50	2.22***	1.62	3.04
Mexico City	1.11	0.82	1.5	1.09	0.80	1.48	1.13	0.81	1.57	1.06	0.73	1.55
Foreign-born US Hispanics	1.26	0.83	1.89	1.17	0.76	1.80	1.09	0.69	1.71	1.09	0.68	1.76
*Depression*												
Havana	2.36***	1.81	3.07	2.37***	1.81	3.10	1.99***	1.48	2.66	2.16***	1.59	2.95
Mexico City	1.51**	1.12	2.04	1.52**	1.11	2.07	1.55**	1.12	2.16	1.50*	1.03	2.19
Foreign-born US Hispanics	1.48*	1.05	2.08	1.36	0.95	1.95	1.36	0.93	2.01	1.49*	1.00	2.21
*Mobility limitations*												
Havana	2.20***	1.75	2.75	2.14***	1.70	2.68	2.06***	1.60	2.64	2.21***	1.68	2.91
Mexico City	1.72**	1.23	2.40	1.65**	1.17	2.32	1.65**	1.44	2.38	1.64*	1.08	2.47
Foreign-born US Hispanics	2.59***	1.81	3.71	2.41***	1.65	3.52	2.22***	1.48	3.34	2.23***	1.47	3.39

Model I: Controlling for age

Model II: Controlling for age, education, and income

Model III: Controlling for age, education, income, partnership, and number of children

Model IV: Controlling for age, education, income, partnership, number of children, and smoking

Significance levels: *p < 0.05; **p < 0.01, ***p < 0.001

Women in Havana had odds of reporting poor health that were around twice as high as those of men (OR 1.84 CI 1.34; 2.53). In this sample, the relative gender gaps in the prevalence of poor SRH increased only slightly after family characteristics and smoking were controlled for (OR 2.00 CI 1.38; 2.92). In the Mexico City sample, the odds ratios became slightly smaller after the model was adjusted for SES and family characteristics and were no longer significant after smoking was added to the model. The odds of reporting poor health were similar among women and men in the US Hispanic sample.

For ADL disability, the initial relative gender gaps in the Havana sample (OR 1.75 CI 1.3; 2.27) decreased after adjusting for SES (OR 1.66 CI 1.27; 2.17) and increased when family characteristics and smoking were included in the model (OR 2.22 CI 1.62; 3.04). The gender gaps remained non-significant for the Mexico City sample and for the foreign-born Hispanics in the USA when all additional control variables were included.

Finally, in the Havana sample, the odds of depression were twice as high among women than among men (OR 2.36 CI 1.81; 3.07). Including additional covariates revealed that family characteristics (OR 1.99 CI 1.48; 2.66) and, to a lesser extent, smoking (OR 2.16 CI 1.59; 2.95) played a potentially important role in explaining gender differences in depression. In the Mexico City sample, women had higher odds of depression than men, and this ratio remained at almost the same level after adjusting for all control variables (from OR 1.51 CI 1.12; 2.04 to OR 1.50 CI 1.03; 2.19). Among the foreign-born Hispanics in the USA, gender differences in depression were marginally statistically significant in models I and IV.

In the Havana sample, the odds of having mobility limitations were twice as high among women than among men (OR 2.20 CI 1.75; 2.75). The relative gender gap remained almost unchanged when SES, family characteristics, and smoking behaviour were controlled for (OR 2.21 CI 1.68; 2.91). Similar patterns were observed for the Mexico City sample with a consistent female disadvantage in mobility limitations. The relative gender differentials in mobility limitations were the largest among the foreign-born Hispanics in the USA: women were more than twice as likely to have mobility limitations compared to men (OR 2.59 CI 1.81; 3.71). The inclusion of all covariates just marginally reduced this disadvantage (OR 2.23 CI 1.47; 3.39).

In summary, the relative gender differentials in the prevalence of the four health problems were systematically pronounced and statistically significant only in the Havana sample. In the foreign-born Hispanic sample, gender differences were apparent for mobility limitations and depression. The female respondents in Mexico City had a clear health disadvantage in poor SRH, mobility limitations, and depression, although not in ADL disability. Additional accounting for potential confounders resulted in only small changes in the relative female disadvantage in all four health domains and in all three samples.

## Discussion

The aim of the present study was to compare gender differentials in health in Havana (Cuba) with those in Mexico City (Mexico) and in the US Hispanic population. Our findings indicate that both the absolute and, in particular, the relative gender gaps in four major health domains were consistently more pronounced in the Havana sample than in the samples for Mexico City and foreign-born Hispanics in the USA. The gender gap observed among the Havana respondents persisted even after controlling for SES, family characteristics, and smoking. Although the larger relative differentials in Cuba can be attributed to the much lower overall (national) and gender-specific prevalence of poor health, the present study shows that there was a strikingly large female disadvantage in health in Havana with respect to both absolute and relative gender gaps.

Our findings suggest that although Cuba has been maintaining high levels of gender and social equity (including universal access to health care) and can be regarded as one of the longevity vanguards in Latin America and Caribbean region, women in Havana (aged 60+) bear a disproportionate burden of ill health, as tends to be the case for women in much less equitable societies. This pattern may also be a consequence of the major causes of death shifting towards the stage when chronic age-related diseases are dominant.

The Cuban experience suggests that ensuring universal access to basic medical care, which is very efficient, for example, in the prevention of infectious diseases (Cooper 2006; Macdonald et al. 2006b), may be not sufficient to address recently emerging health threats that require modern and costly medical technologies for early diagnosis and treatment. Importantly, older adults can be considered a vulnerable group in the Cuban health system due to the scarcity of health care resources, which are predominantly allocated to provide care for children and women of childbearing age (Da Silva Coqueiro et al. 2010). Our study provides indirect evidence that Cuba has limited health care resources for the older population, as the treatments and procedures needed by older patients tend to be expensive and sophisticated and often involve more risk due to the presence of comorbid conditions. Furthermore, Verstraeten et al. (2016) showed that women in the Caribbean were generally more disadvantaged than men because of their poorer working conditions, higher risk of experiencing sexual and physical violence, and disproportionately large care burdens as heads of single-parent families. Our study has highlighted the disadvantages of Cuban women in several key health dimensions and points towards a contradiction between the formally declared high levels of gender and social equality under communism (Sarmiento 2010) and the actual gender gaps observed.

The gender gaps in health in Mexico City and among the foreign-born Hispanics in the USA were less consistent. The finding of no gender gaps in poor SRH and ADL disability among foreign-born Hispanics in the USA was particularly surprising.

Male-dominated migration of Latin Americans to the USA continues to occur despite the increase in female migrants in recent years (Riosmena and Massey 2012). Because our study population included only foreign-born Hispanics in the USA aged 60+, most of them are likely to have migrated years ago. Thus, the initial health advantage of Hispanic immigrants to the USA may have decreased over time because of adverse effects of hazardous jobs following immigration to the USA and poorer lifestyles, which are more prevalent among men than among women (Antecol and Bedard 2006). The existing literature has also shown that the health of US Hispanics tends to differ substantially by their generational status (foreign-born versus US-born), place of residence, and how quickly they assimilate the American lifestyle (Escarce et al. 2006). While we were not able to include some of these measurements, our results provided some indications that lower SES among women might be an important contributor to the disadvantage in mobility limitations observed among the foreign-born Hispanic women.

Our study also explored the question of whether gender differentials in health can be explained by differences in the socio-economic and behavioural characteristics of men and women. We found that controlling for differences in socio-economic status, family characteristics, and smoking behaviour did not produce substantial changes in the originally observed age-adjusted relative gender differences.

Our findings that there were no female disadvantages in SRH and ADL disability partially contradict previous results that reported gender differences among Mexicans aged 60+ in the prevalence of diabetes, depression, anaemia, and malnutrition (Wheaton and Crimmins 2015). A possible explanation for these inconsistent findings is related to where the respondents live, as the place of residence may be an important component of the impact of socio-economic characteristics on gender differences in health. As almost all of the population of Mexico City live in urban areas, gender differences may be less evident there.

Accounting for family characteristics did not modify the magnitude of gender differences in most health outcomes in all three study populations, but they may have played an important role in the relationship between gender and depression in the Havana sample. Controlling for current partnership and number of children slightly reduced the gender gap in depression, which is in line with the social support literature suggesting that emotional support has a positive effect on mental health (Kawachi and Berkman 2001).

Finally, we explored the potential role of smoking in explaining the gender gap in the selected health characteristics. In the Havana sample, gender differences in poor SRH, ADL disability, and mobility limitation slightly increased after adjusting for smoking behaviour, while in the Mexico City and the foreign-born US Hispanic samples, gender differences remained almost unchanged. The differences in effects of smoking may be explained by the relatively high prevalence of women who smoke in Havana in comparison with Mexico City and the foreign-born US Hispanics (See Supplementary Table 1).

### Strengths and limitations

The major strength of our study is the extension of research on gender inequalities in health to Latin American populations and the consideration of various health outcomes. The study also includes three settings with very different gender equities (Cuba and Mexico), selection into old age (US Hispanics), and health care access.

However, this study comes with some limitations. SABE was conducted only in the very big cities of Havana and Mexico City. Therefore, the data do not represent the entire Cuban or Mexican populations. It is noteworthy that the health care infrastructure, particularly for the 60+ population, appears to be more advanced in urban areas than in rural areas. In Cuba, Havana has the highest concentration of the population aged 60+ (19.5% for 2009), and the living circumstances of older people are particularly challenging due to a lack of services and poor housing conditions. Thus, the (older) population in Havana may not differ from the Cuban population as a whole with respect to health or quality of life (Coyula 2010).

In Mexico City, the situation is not much different. In a newspaper article, Cruz-Flores (2018) reported that the capital is one of the richest areas of the country but suffers from an uneven distribution of health care providers (i.e. doctors and nurses) and infrastructure within the city itself. The hospital infrastructure in particular is lagging behind, as most of Mexico City’s hospitals were built over 60 years ago and are not equipped to deal with the chronic conditions that affect the population of the capital, or the infectious diseases that are common in the peripheral neighbourhoods of the city.

Furthermore, our results obtained from the HRS sample (2000) may not be generalisable to the whole population of US Hispanics, as the characteristics of this population, like place of residence and country of origin, have become more diverse in recent years (Ennis et al. 2011).

Another key concern in this cross-national comparison study is the comparability of the SABE and the HRS data. Although the data on the Havana and Mexico City populations were collected using the same data collection format, the HRS applied a different methodology, which could interfere with the comparison of health disparities across populations within each gender. Moreover, the comparability of mobility limitations across settings can be hindered by differences in public or housing environments, for example, the presence of ramps, elevators, and adequately designed stairs. However, the aim of the present study was not to provide a cross-country comparison of health, but to examine gender differences in health. The latter comparison is to be likely less sensitive to cross-country differences in data collection instruments, response patterns, and public or housing environments. Supporting this proposition, recent research found apparent cross-country differences but no clear gender differences in reporting of health (Jürges 2007; Oksuzyan et al. 2019; Spitzer and Weber 2019). These findings suggest that the comparison of self-reported health measures between genders across countries should be credible.

Moreover, SABE and HRS included only non-institutionalised populations. Since widowhood is more prevalent among women than men (Dupre et al. 2009), a man in need of care is more likely to be living in the community with his female spouse, while a woman in need of care is more likely to be living in a nursing home or other long-term care facility (Hurd et al. 2014). Thus, gender differences in more serious disabilities can be underestimated in community-based samples (Kelfve et al. 2013). However, we do not expect that underestimation occurred in our study, given that for cultural and social reasons, institutionalisation is a much less common practice among Cubans, Mexicans, and US Hispanics than it is among the populations of high-income countries (Angel et al. 2014).

Finally, we cannot exclude the possibility that some respondents could misinterpret or misunderstand the questions posed to them or misinform, voluntarily or not, by incorrectly assessing their condition (Rosenman et al. 2011).

## Conclusion

The present study shows that both the absolute and relative gender gaps in self-rated health, ADL disability, depression, and mobility limitations were consistently more pronounced among the Havana respondents than among the Mexico City sample and the foreign-born Hispanics in the USA, except for mobility limitations. It is unknown how the magnitude in gender differences in these health domains developed in the last two decades. Thus, there is an urgent need to collect longitudinal data on subjective and objective health measures in order to investigate health trends in Cuba and in other Latin American and Caribbean countries, including for the young and the middle-aged populations, and to collect data on causes of death in order to examine trends and gender differences in mortality in these countries. Finally, it will be particularly important for researchers to track how recent political changes in the Cuba–USA relationship, including their economic dimensions, will affect the functioning of the health care system in Cuba, which needs to be modernised and given more resources to combat ageing-related health threats, including those that disproportionately affect women.

## Electronic supplementary material

Below is the link to the electronic supplementary material.

Supplementary material 1 (DOCX 57 kb)

## Footnotes

1 The National Center for Health Statistics also provides for the years 2006 to 2016 data on LE for US Hispanics (NCHS 2017). Numbers are available under https://www.cdc.gov/nchs/data/hus/2017/015.pdf and are not shown in the figures.
