# Supplementary material for: Gender differences in health in Havana versus in Mexico City and in the US Hispanic population
Source: Eur J Ageing. 2020 Mar 16;18(2):217–26. doi: 10.1007/s10433-020-00563-w (PMC8217427; doi:10.1007/s10433-020-00563-w)
Supplement: Supplementary file 1 — Supplementary material 1 (DOCX 57 kb) [file 10433_2020_563_MOESM1_ESM.docx]

**Supplementary Materials**

**Gender Differences in Health in Havana versus in Mexico City and in the U.S. Hispanic Population**

***European Journal of Ageing***

Mine Kühn^a*^, Carlos Díaz-Venegas^a^, Domantas Jasilionis^ab^, Anna Oksuzyan^a^,

^a^ Max Planck Institute for Demographic Research, Konrad-Zuse Straße 1, 18057 Rostock, Germany.

^b^ Demographic Research Centre, Vytautas Magnus University, Jonavos str. 66-212, LT-44191 Kaunas, Lithuania

^*^Corresponding author:

Email: kuehn@demogr.mpg.de

Table 1. Descriptive table of control variables

Table 2. Relative gender differentials (odds ratios) in Poor Self-Rated-Health

Table 3. Relative gender differentials (odds ratios) in ADL-Disability

Table 4. Relative gender differentials (odds ratios) in Mobility Limitation

Table 5. Relative gender differentials (odds ratios) in Depression

**Table 1. Descriptive table of control variables**

|  | **Havana** | | | **Mexico City** | | | **U.S. Hispanics** | | |
| --- | --- | --- | --- | --- | --- | --- | --- | --- | --- |
|  | N | Men | Women | N | Men | Women | N | Men | Women |
| **Age** |  |  |  |  |  |  |  |  |  |
| 60-64 |  | 30.93 | 22.06 |  | 31.76 | 30.00 |  | 31.25 | 32.71 |
| 65-69 |  | 23.59 | 19.63 |  | 25.25 | 24.86 |  | 23.75 | 20.25 |
| 70-74 |  | 16.53 | 19.38 |  | 18.34 | 18.44 |  | 15.83 | 13.08 |
| 75 and older |  | 28.95 | 38.22 |  | 24.65 | 25.98 |  | 29.17 | 33.96 |
| **Education** |  |  |  |  |  |  |  |  |  |
| No school/Incomplete Primary |  | 4.24 | 5.29 |  | 16.96 | 23.11 |  | 46.03 | 42.37 |
| Completed Primary |  | 43.08 | 57.13 |  | 59.57 | 57.70 |  | 12.13 | 16.20 |
| Some Secondary and more |  | 52.68 | 37.58 |  | 23.47 | 19.19 |  | 41.84 | 41.43 |
| **Perceived current income** |  |  |  |  |  |  |  |  |  |
| Sufficient |  | 22.38 | 19.60 |  | 52.2 | 50.00 |  |  |  |
| Insufficient |  | 77.62 | 80.40 |  | 47.8 | 50.00 |  |  |  |
| **Monthly income** |  |  |  |  |  |  |  |  |  |
| No income or indebted |  |  |  |  |  |  |  | 7.51 | 9.97 |
| Less than $10,000 USD |  |  |  |  |  |  |  | 49.58 | 71.02 |
| Between $10,000 and $19,999 USD | |  |  |  |  |  |  | 23.33 | 13.40 |
| $20,000 USD or more |  |  |  |  |  |  |  | 19.58 | 5.61 |
| **Current partnership** |  |  |  |  |  |  |  |  |  |
| Married/cohabitated |  | 64.27 | 21.39 |  | 76.09 | 38.94 |  | 75.63 | 43.13 |
| Separated/divorced/single |  | 20.76 | 31.76 |  | 7.10 | 18.78 |  | 11.34 | 23.43 |
| Widowed |  | 14.83 | 46.78 |  | 16.77 | 42.03 |  | 13.03 | 33.44 |
| **Number of children** |  |  |  |  |  |  |  |  |  |
| 0 |  | 11.86 | 14.29 |  | 3.76 | 6.62 |  | 3.75 | 9.66 |
| 1 |  | 14.41 | 17.54 |  | 3.96 | 5.68 |  | 8.75 | 10.59 |
| 2 |  | 27.26 | 25.23 |  | 8.51 | 8.11 |  | 14.58 | 16.51 |
| 3 and more |  | 46.47 | 42.94 |  | 83.76 | 79.59 |  | 72.92 | 63.24 |
| **Smoking** |  |  |  |  |  |  |  |  |  |
| Smoke present time |  | 46.89 | 20.57 |  | 29.76 | 7.71 |  | 16.67 | 5.61 |
| Smoked before |  | 29.94 | 14.80 |  | 40.87 | 13.67 |  | 11.67 | 7.17 |
| Never smoked |  | 23.16 | 64.63 |  | 29.37 | 78.62 |  | 71.67 | 87.22 |
| **Total Sample** | 1905 | 708 | 1197 | 1247 | 740 | 507 | 561 | 240 | 321 |
| *Note: Subtotals may not be equal to total sample due to missing cases.* | | | | |  |  |  |  |  |

| **Table 2. Poor Self-Rated-Health** |  |  |  |  |  |  |  |  |  |  |  |  |
| --- | --- | --- | --- | --- | --- | --- | --- | --- | --- | --- | --- | --- |
|  | **Havana** | | | | **Mexico City** | | | | **U.S. Hispanics** | | | |
|  | **I** | **II** | **III** | **IV** | **I** | **II** | **III** | **IV** | **I** | **II** | **III** | **IV** |
| Women | 1.84*** | 1.84*** | 1.85** | 2.00*** | 1.47* | 1.40* | 1.44* | 1.43 | 1.21 | 0.99 | 1.03 | 1.14 |
| Age (ref.: 60-64) |  |  |  |  |  |  |  |  |  |  |  |  |
| 65-69 | 1.37 | 1.32 | 1.29 | 1.29 | 1.14 | 1.17 | 1.18 | 1.17 | 0.93 | 0.63 | 0.65 | 0.65 |
| 70-74 | 0.93 | 0.89 | 0.91 | 0.92 | 1.13 | 1.14 | 1.16 | 1.13 | 0.98 | 0.72 | 0.81 | 0.64 |
| 75+ | 0.91 | 0.81 | 0.84 | 0.86 | 1.15 | 1.19 | 1.22 | 1.14 | 0.93 | 0.50* | 0.59 | 0.60 |
|  |  |  |  |  |  |  |  |  |  |  |  |  |
| Education (ref.: Secondary or More) |  |  |  |  |  |  |  |  |  |  |  |  |
| No Education / Incomplete Primary |  | 2.26** | 1.97* | 1.94* |  | 3.36*** | 3.44*** | 3.48*** |  | 2.16** | 2.10* | 2.04* |
| Completed Primary |  | 1.26 | 1.20 | 1.19 |  | 2.30** | 2.32*** | 2.38*** |  | 1.12 | 1.12 | 1.08 |
|  |  |  |  |  |  |  |  |  |  |  |  |  |
| Perception of Income (SABE)/Income ([HRS](#_ENREF_19)) (ref: Sufficient/ > 20,000 USD) |  |  |  |  |  |  |  |  |  |  |  |  |
| Insufficient (SABE)/No Income or Indebted |  | 1.36 | 1.35 | 1.34 |  | 1.81*** | 1.81*** | 1.81*** |  | 1.71 | 1.66 | 1.67 |
| <10,000 USD ([HRS](#_ENREF_19)) |  |  |  |  |  |  |  |  |  | 5.40** | 5.10** | 4.99* |
| 10,000 -20,000 USD ([HRS](#_ENREF_19)) |  |  |  |  |  |  |  |  |  | 2.02 | 2.02 | 2.01 |
|  |  |  |  |  |  |  |  |  |  |  |  |  |
| Partnership (ref.: Married, Cohabited) |  |  |  |  |  |  |  |  |  |  |  |  |
| Separated, Divorced, Single |  |  | 1.09 | 1.09 |  |  | 0.97 | 0.99 |  |  | 1.50 | 1.38 |
| Widowed |  |  | 0.99 | 0.99 |  |  | 0.88 | 0.88 |  |  | 0.77 | 0.80 |
| Number of Children (ref.: 1/2) |  |  |  |  |  |  |  |  |  |  |  |  |
| 0 |  |  | 1.14 | 1.12 |  |  | 0.92 | 0.92 |  |  | 1.34 | 1.38 |
| 3+ |  |  | 1.57** | 1.55** |  |  | 0.81 | 0.82 |  |  | 1.33 | 1.31 |
| Smoking (ref.: never smoked) |  |  |  |  |  |  |  |  |  |  |  |  |
| Smoke now |  |  |  | 1.20 |  |  |  | 0.65 |  |  |  | 1.52 |
| Smoked before |  |  |  | 1.27 |  |  |  | 1.32 |  |  |  | 2.68* |
| Constant | 0.09*** | 0.06*** | 0.05*** | 0.04*** | 0.17*** | 0.06*** | 0.07*** | 0.07*** | 0.18*** | 0.05*** | 0.04*** | 0.03*** |
| Significance levels: * p<0.05, ** p<0.01, *** p<0.001. | | | | |  |  |  |  |  |  |  |  |

| **Table 3. ADL-Disability** | | | | | | | | | | | | |
| --- | --- | --- | --- | --- | --- | --- | --- | --- | --- | --- | --- | --- |
|  | **Havana** | | | | **Mexico City** | | | | **U.S. Hispanics** | | | |
|  | **I** | **II** | **III** | **IV** | **I** | **II** | **III** | **IV** | **I** | **II** | **III** | **IV** |
| Women | 1.75*** | 1.66*** | 1.86*** | 2.22*** | 1.11 | 1.09 | 1.13 | 1.07 | 1.26 | 1.17 | 1.09 | 1.09 |
| Age (ref.: 60-64) |  |  |  |  |  |  |  |  |  |  |  |  |
| 65-69 | 1.01 | 0.96 | 0.98 | 0.98 | 1.27 | 1.31 | 1.32 | 1.32 | 1.08 | 0.92 | 0.89 | 0.84 |
| 70-74 | 1.01 | 0.92 | 0.98 | 1.02 | 1.67* | 1.81* | 1.82* | 1.81* | 1.09 | 1.00 | 1.14 | 0.93 |
| 75+ | 2.84*** | 2.50*** | 2.85*** | 3.04*** | 3.79*** | 3.60*** | 3.70*** | 3.65*** | 1.50 | 1.10 | 1.13 | 1.06 |
|  |  |  |  |  |  |  |  |  |  |  |  |  |
| Education (ref.: Secondary or More) |  |  |  |  |  |  |  |  |  |  |  |  |
| No Education / Incomplete Primary |  | 1.90* | 1.73* | 1.70* |  | 2.28** | 2.30** | 2.28** |  | 1.61* | 1.66* | 1.69* |
| Completed Primary |  | 1.43** | 1.41* | 1.38* |  | 1.84** | 1.85** | 1.82* |  | 0.71 | 0.70 | 0.69 |
|  |  |  |  |  |  |  |  |  |  |  |  |  |
| Perception of Income (SABE)/Income ([HRS](#_ENREF_19)) (ref: Sufficient/ > 20,000 USD) |  |  |  |  |  |  |  |  |  |  |  |  |
| Insufficient (SABE)/No Income or Indebted |  | 1.45* | 1.43* | 1.42* |  | 1.52** | 1.53** | 1.53** |  | 1.87 | 1.93 | 1.99 |
| <10,000 USD ([HRS](#_ENREF_19)) |  |  |  |  |  |  |  |  |  | 2.23 | 2.13 | 2.12 |
| 10,000 -20,000 USD ([HRS](#_ENREF_19)) |  |  |  |  |  |  |  |  |  | 1.25 | 1.29 | 1.27 |
|  |  |  |  |  |  |  |  |  |  |  |  |  |
| Partnership (ref.: Married, Cohabited) |  |  |  |  |  |  |  |  |  |  |  |  |
| Separated, Divorced, Single |  |  | 0.96 | 0.96 |  |  | 0.86 | 0.87 |  |  | 1.79* | 1.93* |
| Widowed |  |  | 0.72 | 0.73 |  |  | 0.92 | 0.92 |  |  | 1.13 | 1.26 |
| Number of Children (ref.: 1/2) |  |  |  |  |  |  |  |  |  |  |  |  |
| 0 |  |  | 1.18 | 1.15 |  |  | 0.90 | 0.89 |  |  | 1.68 | 1.70 |
| 3+ |  |  | 1.40* | 1.38* |  |  | 0.86 | 0.86 |  |  | 1.08 | 1.11 |
| Smoking (ref.: never smoked) |  |  |  |  |  |  |  |  |  |  |  |  |
| Smoke now |  |  |  | 1.48* |  |  |  | 0.92 |  |  |  | 0.65 |
| Smoked before |  |  |  | 1.73** |  |  |  | 0.87 |  |  |  | 2.85** |
| Constant | 0.10*** | 0.07*** | 0.06*** | 0.04*** | 0.12*** | 0.06*** | 0.07*** | 0.07*** | 0.47*** | 0.25** | 0.20*** | 0.19*** |
| Significance levels: * p<0.05, ** p<0.01, *** p<0.001. | |  |  |  |  |  |  |  |  |  |  |  |

| **Table 4. Mobility Limitation** | | | | | | | | | | | | |
| --- | --- | --- | --- | --- | --- | --- | --- | --- | --- | --- | --- | --- |
|  | **Havana** | | | | **Mexico City** | | | | **U.S. Hispanics** | | | |
|  | **I** | **II** | **III** | **IV** | **I** | **II** | **III** | **IV** | **I** | **II** | **III** | **IV** |
| Women | 2.20*** | 2.14*** | 2.06*** | 2.21*** | 1.72** | 1.65** | 1.65** | 1.64* | 2.59*** | 2.41*** | 2.22*** | 2.23*** |
| Age (ref.: 60-64) |  |  |  |  |  |  |  |  |  |  |  |  |
| 65-69 | 0.99 | 0.99 | 0.95 | 0.94 | 1.06 | 1.14 | 1.14 | 1.14 | 1.60 | 1.25 | 1.30 | 1.24 |
| 70-74 | 0.89 | 0.88 | 0.86 | 0.87 | 1.06 | 1.15 | 1.16 | 1.15 | 2.19** | 1.84* | 2.05* | 1.62 |
| 75+ | 2.03*** | 1.94*** | 1.91*** | 1.92*** | 2.52** | 2.43*** | 2.47** | 2.42** | 3.19*** | 2.09** | 2.08** | 1.99** |
|  |  |  |  |  |  |  |  |  |  |  |  |  |
| Education (ref.: Secondary or More) |  |  |  |  |  |  |  |  |  |  |  |  |
| No Education / Incomplete Primary |  | 1.62 | 1.45 | 1.46 |  | 1.60 | 1.60 | 1.61 |  | 1.91** | 1.82** | 1.82** |
| Completed Primary |  | 1.18 | 1.12 | 1.12 |  | 1.51* | 1.51* | 1.52* |  | 1.22 | 1.18 | 1.18 |
|  |  |  |  |  |  |  |  |  |  |  |  |  |
| Perception of Income (SABE)/Income ([HRS](#_ENREF_19)) (ref: Sufficient/ > 20,000 USD) |  |  |  |  |  |  |  |  |  |  |  |  |
| Insufficient (SABE)/No Income or Indebted |  | 1.08 | 1.09 | 1.10 |  | 1.36 | 1.36 | 1.36 |  | 1.28 | 1.31 | 1.37 |
| <10,000 USD ([HRS](#_ENREF_19)) |  |  |  |  |  |  |  |  |  | 2.69** | 2.54** | 2.67** |
| 10,000 -20,000 USD ([HRS](#_ENREF_19)) |  |  |  |  |  |  |  |  |  | 2.07* | 2.03* | 2.12* |
|  |  |  |  |  |  |  |  |  |  |  |  |  |
| Partnership (ref.: Married, Cohabited) |  |  |  |  |  |  |  |  |  |  |  |  |
| Separated, Divorced, Single |  |  | 1.10 | 1.11 |  |  | 1.08 | 1.09 |  |  | 1.76* | 1.80* |
| Widowed |  |  | 1.20 | 1.21 |  |  | 0.97 | 0.97 |  |  | 1.36 | 1.46 |
| Number of Children (ref.: 1/2) |  |  |  |  |  |  |  |  |  |  |  |  |
| 0 |  |  | 0.84 | 0.83 |  |  | 0.96 | 0.96 |  |  | 1.34 | 1.33 |
| 3+ |  |  | 1.33* | 1.32* |  |  | 1.02 | 1.02 |  |  | 1.28 | 1.30 |
| Smoking (ref.: never smoked) |  |  |  |  |  |  |  |  |  |  |  |  |
| Smoke now |  |  |  | 1.12 |  |  |  | 0.89 |  |  |  | 0.76 |
| Smoked before |  |  |  | 1.38* |  |  |  | 1.07 |  |  |  | 2.75** |
| Constant | 1.91*** | 1.68** | 1.50* | 1.29 | 4.03*** | 2.55*** | 2.49** | 2.50** | 0.45*** | 0.20*** | 0.15*** | 0.14*** |
| Significance levels: * p<0.05, ** p<0.01, *** p<0.001. | |  |  |  |  |  |  |  |  |  |  |  |

| **Table 5. Depression** | | | | | | | | | | | | |
| --- | --- | --- | --- | --- | --- | --- | --- | --- | --- | --- | --- | --- |
|  | **Havana** | | | | **Mexico City** | | | | **U.S. Hispanics** | | | |
|  | **I** | **II** | **III** | **IV** | **I** | **II** | **III** | **IV** | **I** | **II** | **III** | **IV** |
| Women | 2.36*** | 2.37*** | 1.97*** | 2.16*** | 1.51** | 1.52** | 1.55** | 1.50* | 1.48* | 1.36 | 1.36 | 1.49* |
| Age (ref.: 60-64) |  |  |  |  |  |  |  |  |  |  |  |  |
| 65-69 | 1.42* | 1.44* | 1.32 | 1.36 | 1.10 | 1.18 | 1.18 | 1.17 | 0.85 | 0.77 | 0.81 | 0.81 |
| 70-74 | 1.21 | 1.20 | 1.07 | 1.12 | 1.26 | 1.30 | 1.30 | 1.28 | 1.22 | 1.13 | 1.24 | 1.04 |
| 75+ | 0.89 | 0.92 | 0.79 | 0.84 | 1.84** | 2.04*** | 2.06** | 2.00** | 1.69* | 1.37 | 1.49 | 1.53 |
|  |  |  |  |  |  |  |  |  |  |  |  |  |
| Education (ref.: Secondary or More) |  |  |  |  |  |  |  |  |  |  |  |  |
| No Education / Incomplete Primary |  | 1.33 | 1.23 | 1.18 |  | 1.93* | 1.92* | 1.91* |  | 1.45 | 1.33 | 1.30 |
| Completed Primary |  | 0.95 | 0.93 | 0.91 |  | 1.71* | 1.71* | 1.71* |  | 1.14 | 1.10 | 1.07 |
|  |  |  |  |  |  |  |  |  |  |  |  |  |
| Perception of Income (SABE)/Income ([HRS](#_ENREF_19)) (ref: Sufficient/ > 20,000 USD) |  |  |  |  |  |  |  |  |  |  |  |  |
| Insufficient (SABE)/No Income or Indebted |  | 1.91*** | 1.90*** | 1.87*** |  | 2.04*** | 2.05*** | 2.05*** |  | 1.73 | 1.68 | 1.69 |
| <10,000 USD ([HRS](#_ENREF_19)) |  |  |  |  |  |  |  |  |  | 1.93* | 1.85 | 1.82 |
| 10,000 -20,000 USD ([HRS](#_ENREF_19)) |  |  |  |  |  |  |  |  |  | 1.59 | 1.55 | 1.53 |
|  |  |  |  |  |  |  |  |  |  |  |  |  |
| Partnership (ref.: Married, Cohabited) |  |  |  |  |  |  |  |  |  |  |  |  |
| Separated, Divorced, Single |  |  | 1.76** | 1.75** |  |  | 0.85 | 0.86 |  |  | 1.27 | 1.19 |
| Widowed |  |  | 1.58** | 1.56** |  |  | 0.98 | 0.98 |  |  | 1.04 | 1.07 |
| Number of Children (ref.: 1/2) |  |  |  |  |  |  |  |  |  |  |  |  |
| 0 |  |  | 1.63** | 1.61** |  |  | 1.25 | 1.24 |  |  | 1.20 | 1.23 |
| 3+ |  |  | 1.36* | 1.35* |  |  | 1.16 | 1.16 |  |  | 1.42 | 1.40 |
| Smoking (ref.: never smoked) |  |  |  |  |  |  |  |  |  |  |  |  |
| Smoke now |  |  |  | 1.32 |  |  |  | 0.80 |  |  |  | 1.52 |
| Smoked before |  |  |  | 1.03 |  |  |  | 1.06 |  |  |  | 2.27** |
| Constant | 0.12*** | 0.07*** | 0.05*** | 0.05*** | 0.14*** | 0.06*** | 0.05*** | 0.05*** | 0.64*** | 0.36*** | 0.27*** | 0.24*** |
| Significance levels: * p<0.05, ** p<0.01, *** p<0.001. | |  |  |  |  |  |  |  |  |  |  |  |
